# Update on brown tumor of hyperparathyroidism

**DOI:** 10.1590/1806-9282.2024S132

**Published:** 2024-06-07

**Authors:** Alex Guedes, Ricardo Gehrke Becker, Suely Akiko Nakagawa, Aparecida Aguiar Lima Guedes

**Affiliations:** 1 Hospital Santa Izabel, Santa Casa de Misericórdia da Bahia, Orthopedic Oncology Group – Salvador (BA), Brazil.; 2 Universidade Federal do Rio Grande do Sul, Hospital of Clinics of Porto Alegre, Orthopedic Trauma Service – Porto Alegre (RS), Brazil.; 3 Reference Center for Bone Tumors and Sarcomas, A.C. Camargo Cancer Center – São Paulo (SP), Brazil.; 4 Universidade Salvador, Faculty of Medicine – Salvador (BA), Brazil.

## BACKGROUND

On the right and left sides of the thyroid, the parathyroid glands are presented in the form of four nodules in total, two apical and two basal^1^. Topographic variations are common—the parathyroid glands can be located near the larynx or even in the mediastinum, near the thymus^1^. Microscopically, they are made up of two main types of cells, clear and oxyphilic; the former secrete parathyroid hormone (PTH), and the latter have a still obscure function—all are arranged in a chordonal arrangement, interspersed with lobes of fatty tissue^1,2^.

Parathyroid hormone is a calcitonin antagonist that directly acts on renal tubule cells, inhibiting phosphate reabsorption and regulating phosphaturia^1^. In the bones, it acts by stimulating the action of osteoclasts which, by enzymatic action, reabsorb the matrix and solubilize calcium^1^. Therefore, PTH plays a key role in serum calcium homeostasis^1-3^.

Excessive production of PTH^4-10^ may occur due to primary hyperparathyroidism (PHP), phosphate retention, skeletal resistance to PTH, impaired PTH degradation, and altered calcium-PTH feedback regulation in secondary hyperparathyroidism (SHP) or persistent tertiary hyperparathyroidism (THP)^2,3,11,12^. Increased PTH production results in hypercalcemia^3^, due to increased calcium absorption in the intestine, increased renal tubular reabsorption, and increased osteoclastic activity^4,5,11^ which leads to bone demineralization, resulting in microfractures hemorrhage, hemosiderin deposition^13^, and excessive vascular proliferation that give such lesions the characteristic brown staining, justifying the nomenclature brown tumor of hyperparathyroidism (BTH)^4,5^.

Brown tumor of hyperparathyroidism has a female predominance^4,5,14^ in a ratio of 3:18 and increases in frequency with aging (especially after the age of 50 years) and after menopause, which is related to hormonal effects^4,5^. It is very rare before puberty, and its incidence increases with age^2,10^.

Hyperparathyroidism (HP) is a pathology characterized by an increase in PTH secretion despite an increase in calcium in the extracellular fluid^10^. The hormone acts by absorbing the calcium present in the bones through the action of osteoclasts and preventing the reabsorption of phosphate in the glomerular filtrate, which causes phosphaturia and hypophosphatemia^10^. It occurs more frequently in the white breed and is rare in the yellow breed, with an overall incidence of about 20/100,000^6^. In the United States, BTH occurs in less than 2% of all HP patients and is especially associated with the most severe forms of the disease and parathyroid carcinoma. The occurrence of HP in young people should raise the suspicion of hereditary diseases such as multiple endocrine neoplasia (MEN) syndrome^2,5^.

Brown tumor of hyperparathyroidism secondary to PHP is very rare^6,15^—only 2–5% of its carriers have this condition, usually caused by massive PTH secretion^6,12,16,17^. PHP can occur due to parathyroid adenoma^4,5,10,13,16^ (up to 85% of cases)^4,5,10^—benign but metabolically active^4,5^, eventually ectopic lesion^7^; parathyroid carcinoma^4,5,10^—which, although a rare cause of PHP (<1% of cases), presents bone involvement (BTH) more frequently (up to 90% of cases) when compared with benign causes of PHP^4,5^; and hereditary factors (5–10% of cases) such as MEN type 1 (comprises up to 95% of hereditary cases of BTH) and 2A, HP-jaw tumor syndrome, and familial isolated HP that can result in BTH if undiagnosed^2,4^.

Secondary hyperparathyroidism is a frequent result of chronic renal failure (CRF)^5,7,14,16^, particularly in dialysis patients, leading to renal osteodystrophy, a clinical condition that commonly causes BTH^5,7,16^ (present in up to 50% of cases)^5^, with extensive bone marrow osteofibrosis and increased osteoclastic bone resorption^7^. The kidneys are unable to produce calcitriol, which promotes the entry of calcium into the bones. In calcitriol scarcity, PTH levels increase, promoting the removal of calcium from the skeleton. Several factors contribute to this, including bone strength to PTH, increased phosphorus retention, which causes malabsorption of calcium in the gut, and inhibition of 1,25(OH)2D production by increased phosphorus^4^.

Persistent tertiary hyperparathyroidism is characterized by excessive secretion of PTH after long-standing SHP, in which the stimulated parathyroids are no longer in reactive mode but have taken on quasi-autonomous function—not unlike PHP, leading to hypercalcemia^12^. In theory, THP occurs due to the monoclonal expansion of parathyroid cells that have acquired an altered setpoint of their calcium-sensing receptor, causing PTH to continue to be secreted despite high serum calcium levels^12^. Other rare causes of THP include X-linked hypophosphatemic rickets, adult-onset hypophosphatemic rickets (autosomal dominant), and oncogenic osteomalacia^12^.

It is important to distinguish between primary parathyroid disorder, in which there is excessive and incomplete PTH secretion, as occurs in PHP, and physiological situations in which these glands respond to stimuli that lead to increased PTH secretion, as in SHP^12^. From a biochemical point of view, the main difference between primary and SHP is that in the former, there is an increase in serum calcium and a reduction in phosphate^16,17^, and in the latter, there is normocalcemia^12^ and hyperphosphatemia^16^. Although both SHP and THP result from chronic stimulation of PTH secretion, serum calcium is always normal in the former, while it is always elevated in the latter. The distinction between PHP and THP is usually evident to the extent that a clearly definable disorder is present, such as long-standing malabsorptive syndrome or chronic kidney disease (CKD), often after kidney transplantation^2,12^.

Vitamin D deficiency may be associated with elevated PTH^12^.

Drugs such as lithium and thiazide diuretics may be associated with an increase in PTH levels^12^.

## DIAGNOSIS

The diagnosis of BTH is based on clinical manifestations, laboratory tests, imaging evaluation, and anatomopathological study^9,18^. However, as these can be non-specific, it is necessary to maintain a high index of suspicion^9,18^, especially in those patients who do not have a diagnosis of HP^2,18^.

## CLINICAL FINDINGS

Clinically, HP (particularly PHP)^16^ presents as "stones, bones, and groans," where "stones" refer to recurrent kidney stones, "bones" refer to bone pain, loss of bone mass, and fractures, and "groans" describe psychic groans and gastrointestinal symptoms such as vomiting, nausea, peptic ulcers, and pancreatitis^3-5,12^. Other findings include hypercalcemia^5,12^, anorexia^5,10^, bloating^10^, constipation^10^, weight loss^5^, muscle weakness^12^, pruritus^12^, soft tissue or vascular calcifications^12^, polyuria^10^, nocturia^10^, polydipsia^10^, and nephrolithiasis^10,12^.

Brown tumor of hyperparathyroidism is an advanced HP finding^10^. Its clinical findings depend on the lesion's size and location and are nonspecific—some patients are asymptomatic. Bone fragility can lead to fractures^1,7,12,17^ which, in turn, lead to pain and disability^7,12,18^. Injuries that affect the spine may be associated with spinal cord compression. Facial deformities can cause difficulty breathing and food swallowing^7^.

## LABORATORY FINDINGS

Laboratory findings include elevated serum PTH^5,9,11^, elevated serum calcium^5,9,11^, decreased serum phosphate^5^, normal or elevated alkaline phosphatase^4,5^, and elevated urate^4^.

Many studies confirm that the clinical manifestations of HP are worse when there is a deficiency of vitamin D, making its dosage an important part of the screening of suspected vitamin D^14^.

The anatomopathological examination is the gold standard modality for the definitive diagnosis^9,19^ of BTH.

## IMAGING EVALUATION

**Figure 1:**
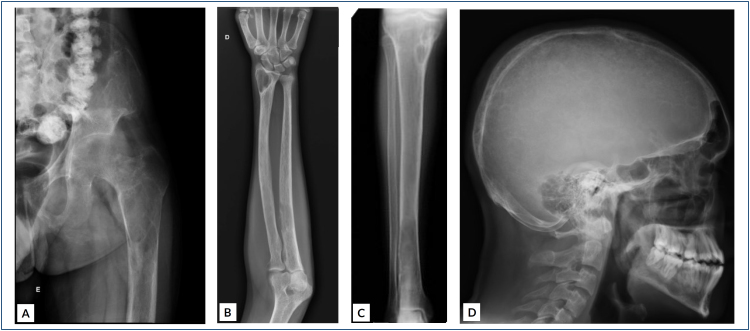
(A–D) A 23-year-old male patient with primary hyperparathyroidism due to parathyroid adenoma presenting disseminated osteolytic bone lesions.

**Figure 2:**
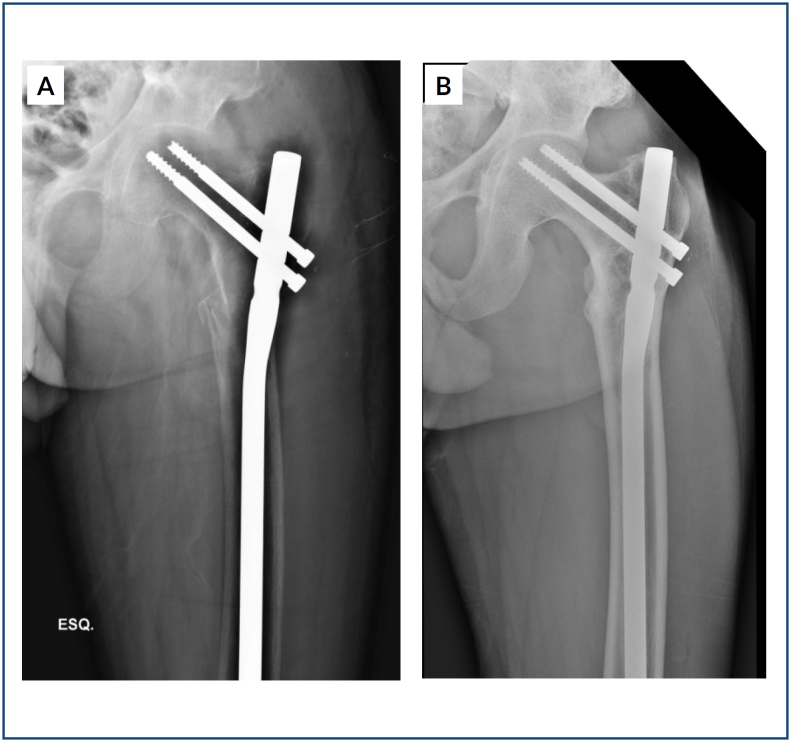
(A, B) A 23-year-old male patient with primary hyperparathyroidism due to parathyroid adenoma, evolving with a pathological fracture through the subtrochanteric bone lesion. (A) Fixation of the fracture with proximal femur nailing. (B) Appearance of the lesion after parathyroidectomy, shortly after fracture fixation.

Brown tumor of hyperparathyroidism can present as diffuse osteopenia^4,5^, osteoporosis^5,6^, bone deformities^4,7^, and circumscribed osteolytic lesions^4-6^ (Figure 1). Bone resorption occurs due to increased osteoclastic activity that affects all bone surfaces in different sites, which may be subperiosteal, intracortical, endosteal, trabecular, subchondral, subligamentous, or subtendinosus^7^. Subperiosteal bone resorption^7,14,18^ is the most striking radiographic feature of HP^7^ and can be observed in the middle phalanges^4,5,7^ (the most sensitive radiographic sign in the diagnosis of BTH)^7^, distal radius^5^, humerus^7^, and clavicle^4-7,14,18^. Subchondral bone resorption is characterized by enlargement or pseudoenlargement of the joint^7^ and occurs in different joints, such as the pubic symphysis and sacroiliac joints, sternoclavicular, and acromioclavicular. Intracortical and endosteal resorption may lead to endosteal clipping findings. The association of trabecular resorption (which causes loss of definition) and granular texture^7^ leads to the "salt and pepper" pattern of the skull^2,4,5,7,9,14^. Subligamentous and subtendinous bone resorption can occur in the ischial tuberosities, trochanters, and insertions of the coracoclavicular ligaments^7^. Bone resorption^7^ can lead to loss of the hard blade of the teeth^4,7,10^ and lesions to the vertebral bodies^4^. BTH^4,5,7,10,14,18^ can occur in the pelvis^4,6-9^, ribs^4,6-9^, long bones^4,6-9^, maxilla^18^, and clavicle^9^. In severe forms of BTH, bone deformities^7^ and insufficiency fractures may occur^7,9,14^ (Figure 2). Excessive resorption of the terminal phalanges can lead to acroosteolysis^7,10^. Severe resorption in the sacroiliac joints can cause pelvic deformities that lead to inability to walk^7^. Thoracic vertebral fractures can lead to an increase in its anteroposterior diameter, leading the thorax to take on a "bell-bottom" shape^7^. Abnormal curvature and vertebral rotation can lead to thoracic deformities^7^.

Multifocal involvement of the skeleton is usually present^4,6,14,20-23^ on radiographs, technetium-99m bone scintigraphy (MDP-99mTc)^4,6,14^, or positron emission tomography-computed tomography (PET-CT).

**Figure 3:**
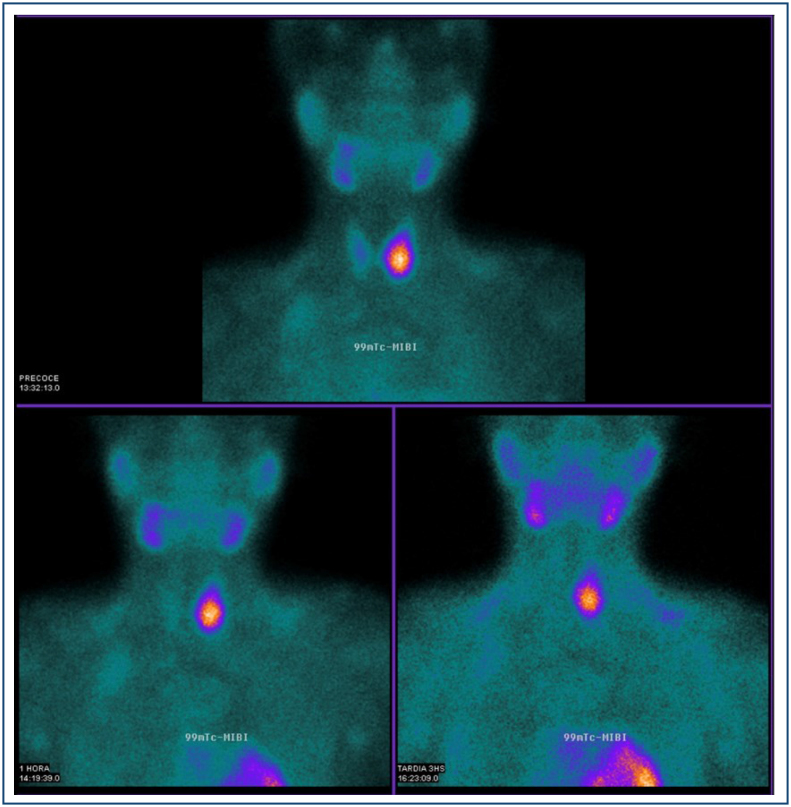
A 23-year-old male patient with a brown tumor of hyperparathyroidism secondary to parathyroid adenoma. Scintigraphy showing hyperuptake of the left parathyroid glands.

Computed tomography (CT)^5,8,24^, MDP-99mTc bone scan^5,8,24^ (Figure 3), and ultrasound^5,6,8^ may be useful for detecting parathyroid gland disorders.

## HISTOPATHOLOGY

Brown tumor of hyperparathyroidism consists of vascularized osteofibrous tissue, devoid of matrix. Microscopically, there is increased resorption of bony trabeculae, forming a "tunneling" or "dissection" pattern. Osteoclastic resorption^4,5,11,18,19^ leads to microfractures and microhemorrhages that progressively produce a small vacuum that becomes confluent with others, making BTH visible macroscopically^3,5,6,8,11,16,18^. Osteoclasts consume the trabecular bone that osteoblasts establish, and this front of reparative bone deposition, followed by further resorption, can expand beyond the usual shape, from bone to the periosteum, and cause bone pain. Involvement of the bones by BTH weakens them, resulting in pathological fractures^4^.

## DIFFERENTIAL DIAGNOSIS

The imaging and histological features of BTH overlap with findings common to different diseases, making differential diagnosis difficult^9,11,14,18^. However, the clinical history of PHP or CRF with SHP usually establishes the diagnosis^4,6^.

It is critical to distinguish BTH from other clinical conditions to avoid unnecessary surgical procedures^18^.

The clinical picture "stones, bones, and groans" can be reproduced in malignant neoplasms such as paraneoplastic syndrome, due to the high levels of PTH-related peptides (PTHrP) that simulate the effect of PTH. In these cases, BTH can be mistaken for bone metastases^12^.

If hypercalcemia is present, the first impression is often of a malignant lesion^14^.

Giant cell tumor of bone^5,8,14,18,25^, aneurysmal bone cyst^5,8,14,25^, simple bone cyst^14^, giant cell reparative granuloma^5,8^, fibrous dysplasia^8^, and non-ossifying fibroma^8^ are included in the differential diagnosis of BTH. It can also be confused with a primary malignant bone tumor^14^ or metastatic disease^5,8,9,14,25^, based on radiographic findings, because it often presents with multiple disseminated osteolytic lesions^5,8,14,25^.

Bone scintigraphy, which has hot spots and/or generally high absorption in PHP, lacks specificity as it can also be seen in a variety of other conditions associated with increased bone metabolism, such as trauma, infections, primary or secondary malignant bone lesions, osteomalacia, Paget's disease, and other osteometabolic diseases^14^.

Positron emission tomography-computed tomography does not reliably distinguish malignant from benign skeletal lesions^14^.

Even histology cannot guarantee a correct diagnosis, due to the large number of lesions with multinucleated giant cells^19^. Among the numerous lesions that present these characteristics on anatomopathological examination^11,14,18,19^, the most challenging differential diagnosis occurs between the giant cell tumor and the BTH^9,11,18^. Other lesions, such as reparative cell granulomas, aneurysmal bone cysts, and some types of osteosarcoma, may present macroscopic and microscopic features similar to BTH^14.18^.

## TREATMENT

Treatment of BTH begins with the management of HP, usually by parathyroidectomy, and should occur after the correction of underlying metabolic issues^9,11^. After parathyroidectomy, most bone disorders resulting from BTH will resolve^2,9,11^.

If surgery is not the best treatment option, medical treatment of hypercalcemia, vitamin D deficiency, and hyperphosphatemia may be performed. Serial evaluation of serum calcium, phosphate, PTH, and vitamin D determines the need for treatment^5^.

Regarding the orthopedic approach to the lesions, some studies point to the previous fixation of the fractures, while others indicate the fixation after parathyroidectomy^15^. Prior treatment of fractures is appropriate in cases where there are severe bone lesions associated with hypercalcemia—surgery should be postponed until the manifestations of hypercalcemia are corrected, avoiding intraoperative adverse events^15^. If parathyroidectomy is defined to be performed prior to fracture fixation, one should be aware of the possibility of "starving bone" syndrome, a condition characterized by rapid, deep, and prolonged hypocalcemia, accompanied by hypophosphatemia and hypomagnesemia. Until hypocalcemia resolves, definitive fixation of fractures should be delayed^2,15^.

## PROGNOSIS

Bone changes constitute a late presentation of HP. Bone involvement in HP has shown a significant decrease in incidence in recent decades (from 80 to only 15%)^5^, constituting a very rare presentation of PHP, especially in developed countries, where serum calcium measurement is routinely performed^14,18^. This fact is attributed to the early detection^4,5,8,14,15^ of asymptomatic cases through the monitoring of serum calcium and the treatment^4,14^ of PH in the early stages of the disease. Proactive therapeutic management has made the manifestation of BTH relatively more common in renal osteodystrophy^14^, and 5% of PH cases develop this condition, which usually indicates prolonged or more severe disease^5^.

Bone lesions resulting from BTH are usually resolved through parathyroidectomy. Proper management of HP results in decreased osteoclastic activity and new bone deposition^2,5^.
